# Editorial of Special Issue “Crosstalk between Depression, Anxiety, and Dementia: Comorbidity in Behavioral Neurology and Neuropsychiatry”

**DOI:** 10.3390/biomedicines9050517

**Published:** 2021-05-06

**Authors:** Masaru Tanaka, László Vécsei

**Affiliations:** 1 MTA-SZTE, Neuroscience Research Group, Semmelweis u. 6, H-6725 Szeged, Hungary; vecsei.laszlo@med.u-szeged.hu; 2 Department of Neurology, Interdisciplinary Excellence Centre, Faculty of Medicine, University of Szeged, Semmelweis u. 6, H-6725 Szeged, Hungary

**Keywords:** depression, anxiety, dementia, Alzheimer’s disease, multiple sclerosis, schizophrenia, stroke, lipid, nutraceutical, diabetes

“Where there is light, there must be shadow, ...”

—Carl Jung

—Haruki Murakami

“Somethings can only be seen in the shadows.”

—Carlos Ruiz Zafon

“The world outside you is only a reflection of the world inside you.”

—unknown

Depression, anxiety, and dementia are spectra of the most common symptoms experienced by patients with a wide range of diseases. The symptoms often concur and frequently wax and wane in the course of the diseases. However, they may serve as prodromal indicators for and may inflict in sequelae to a certain condition. Indeed, depression and anxiety are risk factors for dementia, but they are not just comorbidities or sequelae of dementia. This Special Issue highlights laboratory, clinical, and statistical studies on the crosstalk between depression, anxiety, dementia, Alzheimer’s disease (AD), multiple sclerosis (MS), schizophrenia (SCZ), diabetes mellitus (DM), Down’s syndrome, and/or compulsive disorders, presented by 71 authors and edited by 25 referees, three academic editors, and one editor.

Animal research is one of the essential arenas for laboratory sciences in neuropsychiatry. Kisspeptins (KP) are endogenous neuropeptides with L-arginine and L-phenylalanine motif at the C-terminal (RF-amide peptides), which regulate the reproductive system. The N-terminally truncated octapeptide KP-8 induced anxiety-like behavior, reduced ambulatory activity, and suppressed exploratory locomotion by activating the hypothalamic–pituitary–adrenal (HPA) axis and increasing gamma-aminobutyric acid (GABA) release in the nucleus accumbens in rats [1]. The studies on the triple transgenic mouse model of AD model (3xTg-AD) showed higher mortality rates and HPA axis activation in female mice of 3xTg-AD and the wild type, but worse behavioral and cognitive functions, higher cerebral blood flow, and improved cardiovascular phenotypes only in 3xTg-AD female mice. The authors suggested the presence of a sex-dependent compensatory hemodynamic mechanism, proposing a possible target for interventions of dementia in aging [2].

The linkage between late-life depression (LLD) and AD was explored by resting-state functional magnetic resonance imaging (fMRI) studies analyzing the default mode network (DMN), executive control network, and salience network (SN). The dissociated functional connectivity pattern with increased anterior DMN and decreased posterior DMN was commonly observed in LLD and AD. The DMN connectivity increased in LLD and decreased in AD, but the SN connectivity decreased in LLD and increased in AD. The authors proposed that the similarity of dissociation may be a possible mechanism of association between LLD and AD [3]. Depression is a common sequela to stroke attack. Poststroke depression increased the level of disability and mortality rates regardless of stroke severity and other neuropsychiatric symptoms during the first year of stroke or transient ischemic attack. The authors suggested depression as a prognostic biomarker for cerebrovascular accidents [4].

Plasma protein signatures were explored in patients suffering from major depressive disorder (MDD). Longitudinal liquid chromatography tandem mass spectrometry (LC-MS/MS) analysis showed 63 proteins significantly associated with drug response-time interactions, 21 proteins significantly associated with response term, and 15 proteins significantly correlated with psychiatric measurement indices. The authors proposed the LC-MS/MS analysis of the serum proteins for a predictive and prognostic biomarker for MDD [5].

Animal-assisted intervention (AAI) and prerobot intervention (PRI) are interventional strategies for the elderly with cognitive impairment or dementia. Pooled analysis of AAI and PRI on the behavioral and psychological symptoms of dementia (BPSD) revealed that the interventions induced a beneficial impact on the depression component of BPSD, but not on the component of anxiety or quality of life. Thus, the authors revealed that depression is an interventional target for cognitive impairment and dementia [6]. Mushroom-produced psychedelic prodrug psylocibin was shown to be significantly effective in the treatment of depression and anxiety in patients suffering from life-threatening diseases by meta-analysis. The authors emphasized the importance of psilocybin translational research for the treatment of emotional symptoms, especially for the patients resistant to conventional pharmacotherapy [7].

Depression, anxiety, and dementia are common psychobehavioral symptoms in autoimmune demyelinating MS. The disturbance of reduction-oxidation homeostasis was commonly observed in MS. Monitoring various components of reactive chemical species, oxidative enzymes, antioxidative enzymes, and degradation products, including kynurenines was proposed to build personalized treatment plans for a better quality of life in MS [8].

The disturbance of lipid metabolism is gaining increasing attention in neuropsychiatric diseases and their comorbidities. A case-control study revealed that depression, diabetes mellitus, and older age were associated with an increased likelihood of developing AD, and dyslipidemia treatment reduced the likelihood of developing AD. The authors declared that depression and diabetics are risk factors of dementia, treatment of dyslipidemia reduces the risk of dementia, and ageing is a decisive risk factor of dementia [9]. The status of polyunsaturated G-protein coupled receptor (GPR) 120 and its ligands, polyunsaturated fatty acid (PUFA) concentrations was studied in patients suffering from SCZ. Correlations were observed between the serum fatty acids (FAs) and GPR120 concentration in healthy controls (HCs), but no correlation was found in SCZ. Furthermore, alpha-linolenic acid and docosahexaenoic acid were independently associated with GPR120 concentration in the model adjusted for eicosapentaenoic acid in HCs. The authors concluded that a disturbance of PUFA concentrations may play a role in SCZ pathogenesis [10].

The use of nutraceutical compounds was proposed for the prevention of neurodegenerative diseases. A sugar-like compound inositol plays an important role in insulin signaling, oxidative stress, and neuronal activities. Prophylactic and supplemental use of nutraceutical inositol was suggested to prevent development and progression of cognitive impairments in AD, Down’s syndrome, anxiety, compulsive disorder, and depressive disorder [11].

Depression, anxiety, and dementia are insufferable burdens experienced by patients and conspicuous findings exhibited to physicians. However, light is versatile and sometimes mischievous. The symptoms may not be the parts of the spectrum emitting or reflecting from the underlying conditions. Maybe the manifestations are footprints left by or shadows embodied through a certain pathogenesis. However, shadow is miscellaneous and multifarious. The clinical, laboratory, and statistical studies in this Special Issue successfully cast some gleams of light on the silhouette of depression, anxiety, and dementia in comorbidities. In order to capture the sharper image, our mission continues (https://www.mdpi.com/journal/biomedicines/special_issues/neuropsychiatry_2).
